# The Secret Key Capacity of a Class of Noisy Channels with Correlated Sources

**DOI:** 10.3390/e21080732

**Published:** 2019-07-26

**Authors:** Germán Bassi, Pablo Piantanida, Shlomo Shamai (Shitz)

**Affiliations:** 1 School of Electrical Engineering and Computer Science, KTH Royal Institute of Technology, 100 44 Stockholm, Sweden; 2 CentraleSupélec–French National Center for Scientific Research (CNRS)–Université Paris-Sud, 3 Rue Joliot-Curie, F-91192 Gif-sur-Yvette, France; 3 Montreal Institute for Learning Algorithms (MILA), Université de Montréal, 2920 Chemin de la Tour, Montréal, QC H3T 1N8, Canada; 4 Department of Electrical Engineering, Technion–Israel Institute of Technology, Haifa 32000, Israel

**Keywords:** information-theoretic security, secret key agreement, secret key capacity, wiretap channel, correlated sources

## Abstract

This paper investigates the problem of secret key generation over a wiretap channel when the terminals observe correlated sources. These sources are independent of the main channel and the users overhear them before the transmission takes place. A novel outer bound is proposed and, employing a previously reported inner bound, the secret key capacity is derived under certain less-noisy conditions on the channel or source components. This result improves upon the existing literature where the more stringent condition of degradedness is required. Furthermore, numerical evaluation of the achievable scheme and previously reported results for a binary model are presented; a comparison of the numerical bounds provides insights on the benefit of the chosen scheme.

## 1. Introduction

The wiretap channel, introduced by Wyner [1], is the basic model for analyzing secrecy in wireless communications. In this model, the transmitter, named Alice, wants to communicate reliably with Bob while keeping the transmitted message—or part of it—secret from an eavesdropper, named Eve, overhearing the communication through another channel. Secrecy is characterized by the amount of information that is not leaked, which can be measured by the equivocation rate—the remaining uncertainty about the message at the eavesdropper. The secrecy capacity of the wiretap channel is thus defined as the maximum transmission rate that can be attained with zero leakage. In their influential paper [2], Csiszár and Körner determined the rate-equivocation region of a general broadcast channel with any arbitrary level of security, which also establishes the secrecy capacity of the wiretap channel. These schemes guarantee secrecy by exploiting an artificial random noise that saturates the eavesdropper’s decoding capabilities.

On the other hand, Shannon [3] showed that it is also possible to achieve a positive secrecy rate by means of a *secret key*. Alice and Bob can safely communicate over a noiseless public broadcast channel as long as they share a secret key. The rate of this key, however, must be at least as large as the rate of the message to attain zero leakage. The main question that arises in this scenario is therefore: how do the legitimate users safely share the secret key? The answer is that the users should not communicate the key itself, which would then be compromised. Instead, they should only convey enough information to allow themselves to agree upon a key without disclosing, at the same time, any relevant information about it to the eavesdropper (for further discussion, the reader is referred to [4,5]).

**Figure 1:**
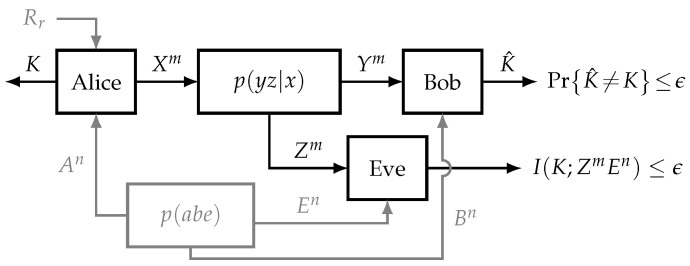
System model for the problem of secret key generation. Every node has access to one of the correlated sources (A,B,E), whereas $R_{r}$ is the local randomness only used by Alice.

In this work, we study the problem of secret key generation over a wiretap channel with correlated sources at each terminal. These sources are assumed to be independent of the main channel and there is no additional public broadcast channel of finite or infinite rate, as seen in Figure 1. It is assumed that each node acquires the *n*-sequence observation of its corresponding source before the communication begins.

### 1.1. Related Work

Maurer [6] and Ahlswede and Csiszár [7] were among the first to study the use of correlated observations available at the legitimate users as a means to agree upon a key. In addition to the correlated observations, the terminals may communicate over a public broadcast channel of infinite capacity to which the eavesdropper has also access. Two models are proposed in [7]: the “source model”, where the users observe correlated random sources controlled by nature, and the “channel model”, where the users observe inputs and outputs of a noisy channel controlled by one of the users. In [8], Csiszár and Narayan studied the first model but assumed that the public broadcast channel has finite capacity and there is a third “helper” node who is not interested in recovering the key but rather helping Alice and Bob. The same authors also analyzed the channel model with only one [9] or with multiple channel inputs [10]. Capacity results are presented in [8,9,10] assuming that there is only one round of communication over the public channel. General inner and outer bounds for both source and channel models with interaction over the public channel were introduced by Gohari and Anantharam [11,12].

More recently, Khisti et al. [13] investigated the situation where there is no helper node, the users communicate over a wiretap channel, and a separate public discussion channel may or may not be available. The simultaneous transmission of a secret message along with a key generation scheme using correlated sources was analyzed by Prabhakaran et al. [14]. They obtained a simple expression that reveals the trade-off between the achievable secrecy rate and the achievable rate of the secret key. The corresponding Gaussian channel with correlated Gaussian sources but independent of the channel components is recently studied in [15]. Closed form expressions for both secret key generation and secret message transmission are derived. On the other hand, Salimi et al. [16] considered simultaneous key generation of two independent users over a multiple access channel with feedback, where each user eavesdrops the other. In addition, the receiver can actively send feedback, through a private noiseless (or noisy) link, to increase the size of the shared keys.

The authors of [13,14,15] did not assume interactive communication, i.e., there is only one round of communication. Salimi et al. [16], however, allowed the end user to respond once through the feedback link. Other authors have analyzed key generation schemes that rely on several rounds of transmissions. Tyagi [17] characterized the minimum communication rate required to generate a maximum-rate secret key with *r* rounds of interactive communication. He showed that this rate is equal to the *interactive common information* (a quantity he introduces) minus the secret key capacity. In his model, two users observe i.i.d. correlated sources and communicate over an error-free channel. Hayashi et al. [18] studied a similar problem but consider general (not necessarily i.i.d.) source sequences of finite length. Their proposed protocol attains the secret key capacity for general observations as well as the second-order asymptotic term of the maximum feasible secret key length for i.i.d. observations. They also proved that the standard one-way communication protocol fails to attain the aforementioned asymptotic result. Courtade and Halford [19] analyzed the related problem of how many rounds of public transmissions are required to generate a specific number of secret keys. Their model assumes that there are *n* terminals connected through an error-free public channel, where each terminal is provided with a number of messages before transmission that it uses to generate the keys. More recently, Boche et al. [20] investigated the computability of the secret key in the source model with only one forward communication. They showed that the corresponding secret key capacity is not Turing computable when the public communication is rate-limited, and consequently there is no algorithm that can simulate or compute the secret key capacity.

As previously mentioned, the focus of the present work is on sources that are independent of the main channel; nonetheless, some works have addressed the general situation of correlated sources and channels. Prior work on secrecy for channels with state include Chen and Vinck’s [21] and Liu and Chen’s [22] analyses of the wiretap channel with state. These works employ Gelfand and Pinsker’s scheme [23] to correlate the transmitted codeword with the channel state at the same time that it saturates the eavesdropper’s decoding capabilities. A single-letter expression of the secrecy capacity for this model is still unknown, although a multi-letter bound was provided by Muramatsu [24] and a novel lower bound is recently reported in [25]. As a matter of fact, the complexity of this problem also lies in the derivation of an outer bound that can handle simultaneously secrecy and channels with state.

To the best of our knowledge, only a handful of works have studied the problem of key generation for channels with state. The previously mentioned result of Prabhakaran et al. [14] is one of these examples. Zibaeenejad [26] analyzed a similar scenario where there is also a public channel of finite capacity between the users and he provides an inner and an outer bound for this model. Although the inner bound is developed for a channel with state, it is possible to apply it to the model used in the present work, i.e., sources independent of the main channel. However, some steps of the proof reported in [26] appear to be obscure and a constraint seems to be missing in the final expression; the resulting achievable rate was recently shown in [27] to be in certain cases unachievable. As a consequence, we decided not to compare our inner bound to this previously reported scheme.

### 1.2. Contributions and Organization of the Paper

In this work, we introduce a novel outer bound (Theorem 2) for the problem of secret key generation over a wiretap channel with correlated sources at each terminal. The correlated sources are assumed to be independent of the main channel and, thanks to a previously reported inner bound (Theorem 1), we obtain the capacity region (Propositions 1–3) whenever the channel and/or source components satisfy the specific *less-noisy* conditions described in Table 1. In contrast, the proposed schemes in [13,14,15,16] are optimal only when the stronger *degradedness* condition holds true for the channel and source components.

The results and tools introduced in this work have connections to ones in a previous work of ours [28], where we studied both the secrecy capacity and the secret key capacity of the wiretap channel with generalized feedback. In [28], we determined some capacity regions for the problem dealt here as a secondary result of the main problem. It is not surprising that, by being the main focus of the present work, the capacity results shown here are more general than those in [28]. Furthermore, we go deeper into the analysis of secret key agreement schemes and we show, in Section 4, the suboptimality of a previously published achievable scheme.

This paper is organized as follows. Section 2 provides some definitions and the previously reported inner bound. In Section 3, we first present the outer bound for the problem of secret key agreement and then we enumerate the cases where we obtain the capacity region. Section 4 illustrates with a binary example the benefit of the present inner bound over a previously reported scheme. Finally, Section 5 summarizes and concludes the work, while some technical proofs are deferred to the appendices.

### 1.3. Notation and Conventions

Throughout this work, we use the standard notation of El Gamal and Kim [29]. Specifically, given two integers *i* and *j*, the expression [i:j] denotes the set {i,i+1,…,j}, whereas for real values *a* and *b*, [a,b] denotes the closed interval between *a* and *b*. We use the notation $x_{i}^{j}=\left(x_{i},x_{i+1},\ldots ,x_{j}\right)$ to denote the sequence of length j−i+1 for 1≤i≤j. If i=1, we drop the subscript for succinctness, i.e., $x^{j}=\left(x_{1},x_{2},\ldots ,x_{j}\right)$. Lowercase letters such as *x* and *y* are mainly used to represent constants or realizations of random variables, capital letters such as *X* and *Y* stand for the random variables in itself, and calligraphic letters such as X and Y are reserved for sets, codebooks, or special functions.

The set of nonnegative real numbers is denoted by $R_{+}$. The probability distribution (PD) of the random vector $X^{n}$, $p_{X^{n}}\left(x^{n}\right)$, is succinctly written as $p(x^{n})$ without subscript when it can be understood from the argument $x^{n}$. Given three random variables *X*, *Y*, and *Z*, if its joint PD can be decomposed as p(xyz)=p(x)p(y|x)p(z|y), then they form a Markov chain, denoted by X —⚬ Y —⚬ Z. The random variable *Y* is said to be *less noisy* than *Z* w.r.t. *X* if I(U;Y)≥I(U;Z) for each random variable *U* such that U —⚬ X —⚬ (Y,Z); this relation is denoted by $Y{⪰}_{X}\;Z$. Entropy is denoted by H(·) and mutual information, I(·;·). The expression ${\left[x\right]}^{+}$ denotes max{x,0}. Given u,v∈[0,1], the function $h_{2}\left(u\right)≜-u\log_{2}u-\left(1-u\right)\log_{2}\left(1-u\right)$ is the binary entropy function and u∗v≜u(1−v)+v(1−u). We denote typical and conditional typical sets by $T_{\delta }^{n}\left(X\right)$ and $T_{\delta }^{n}\left(Y|x^{n}\right)$, respectively.

## 2. Preliminaries

### 2.1. Problem Definition

Consider the *wiretap channel with correlated sources* at every node (A,B,E), as shown in Figure 1. The legitimate users (Alice and Bob) want to agree upon a secret key K∈K while an eavesdropper (Eve) is overhearing the communication. Let A, B, E, X, Y, and Z be six finite sets. Alice, Bob, and Eve observe the random sequences (sources) $A^{n}$, $B^{n}$, and $E^{n}$, respectively, drawn i.i.d. according to the joint distribution p(abe) on A×B×E. Alice communicates with Bob through *m* instances of a discrete memoryless channel with input X∈X and output Y∈Y. Eve is listening the communication through another channel with input X∈X and output Z∈Z. This channel is defined by its transition probability p(yz|x) and it is independent of the sources’ distribution.

**Definition** **1** (Code)**.** *A $\left(2^{nR_{k}},n,m\right)$ secret key code $c_{n}$ for this model consists of:*

- *a key set $K_{n}≜\left[1:2^{nR_{k}}\right]$, where $R_{k}$ is the* rate *of the secret key;*
- *a source of local randomness $R_{r}\in R_{r}$ at Alice;*
- *an encoding function $\varphi :A^{n}\times R_{r}\to X^{m}$;*
- *a key generation function $\psi_{a}:A^{n}\times R_{r}\to K_{n}$; and*
- *a key generation function $\psi_{b}:B^{n}\times Y^{m}\to K_{n}$.*

*The rate of such a code is defined as the number of channel uses per source symbol $\frac{m}{n}$.*

Given a code, let $K=\psi_{a}\left(A^{n},R_{r}\right)$ and $X^{m}=\varphi \left(A^{n},R_{r}\right)$; then, the performance of the $\left(2^{nR_{k}},n,m\right)$ secret key code $c_{n}$ is measured in terms of its average probability of error

(1) $$P_{\;e}\left(c_{n}\right)≜\mathrm{Pr}\;\left\{\psi_{b}\left(B^{n},Y^{m}\right)\neq K|c_{n}\right\},$$

in terms of the information leakage

(2) $$L_{k}\left(c_{n}\right)≜I\left(K;E^{n}Z^{m}|c_{n}\right)\;,$$

and in terms of the uniformity of the keys

(3) $$U_{k}\left(c_{n}\right)≜nR_{k}-H\left(K|c_{n}\right)\;.$$

**Definition** **2** (Achievability)**.** *A tuple $\left(\eta ,R_{k}\right)\in R_{+}^{2}$ is said to be* achievable *for this model if, for every ϵ>0 and sufficiently large n, there exists a $\left(2^{nR_{k}},n,m\right)$ secret key code $c_{n}$ such that*

(4) $$\begin{array}{ccccccccc} \frac{m}{n} & \leq \eta +\epsilon \;, & P_{\;e}\left(c_{n}\right) & \leq \epsilon \;, & L(c_{n}) & \leq \epsilon \;, & \mathrm{and} & U(c_{n}) & \leq \epsilon \;. \end{array}$$

*The set of all achievable tuples is denoted by $R^{⋆}$ and is referred to as the secret key region.*

### 2.2. Inner Bound

The following theorem gives an inner bound on $R^{⋆}$, i.e., it defines the region $R_{\mathrm{in}}\subseteq R^{⋆}$.

**Theorem** **1** ([30], Theorem 2)**.** *A tuple $\left(\eta ,R_{k}\right)\in R_{+}^{2}$ is achievable if there exist random variables U, V, Q, T, and X on finite sets U, V, Q, T, and X, respectively, with joint distribution p(uvqtxyzabe)=p(q|t)p(tx)p(yz|x)p(abe)p(v|a)p(u|v), which verify*

(5) $$R_{k}\leq \eta \left[I(T;Y|Q)-I(T;Z|Q)\right]+I\left(V;B|U\right)-I\left(V;E|U\right)$$

*subject to*

(6a) $$\begin{array}{cc} I(U;A|B) & \leq \eta \;I(Q;Y)\;, \end{array}$$

(6b) $$\begin{array}{cc} I(V;A|B) & \leq \eta \;I(T;Y)\;. \end{array}$$

*Moreover, it suffices to consider sets U, V, Q, and T such that |U|≤|A|+2, |V|≤(|A|+1)(|A|+2), |Q|≤|X|+2, and |T|≤(|X|+1)(|X|+2).*

**Sketch** **of** **Proof.** Alice employs the two-layer description (U,V) to compress the source *A* and she transmits it through the two-layer channel codeword (Q,T). Each layer of the description must fit in the corresponding layer of the channel codeword according to Equation (6). In brief, the encoder randomly picks codewords $u^{n}\left(s_{1}\right)$ from $T_{\delta }^{n}\left(U\right)$ and, for each one, it randomly picks codewords $v^{n}\left(s_{1},s_{2}\right)$ from $T_{\delta }^{n}\left(V|u^{n}\left(s_{1}\right)\right)$. After observing the source sequence $a^{n}$, the encoder selects the indices $\left({\hat{s}}_{1},{\hat{s}}_{2}\right)$ of the codewords that are jointly typical with $a^{n}$. The codewords $u^{n}\left(s_{1}\right)$ and $v^{n}\left(s_{1},s_{2}\right)$ are distributed in bins, i.e., $u^{n}\left(s_{1}\right)\in B_{1}\left(r_{1}\right)$ and $v^{n}\left(s_{1},s_{2}\right)\in {\tilde{B}}_{2}\left(s_{1},r_{2},r_{p}\right)$, and it is the bin indices $\left({\hat{r}}_{1},{\hat{r}}_{2},{\hat{r}}_{p}\right)$ which are transmitted through the noisy channel. The channel codewords $q^{m}\left(r_{1},r_{2}\right)$ are randomly picked from $T_{\delta }^{m}\left(Q\right)$ and, for each $q^{m}\left(r_{1},r_{2}\right)$, the codewords $t^{m}\left(r_{1},r_{2},r_{p},k_{2},r_{f}\right)$ are randomly picked from $T_{\delta }^{m}\left(T|q^{m}\left(r_{1},r_{2}\right)\right)$. In addition to the bin indices from the two-layer description of the source, the encoder uses the noisy channel to transmit a part of the secret key ($k_{2}$), which is protected using a wiretap code; the dummy index $r_{f}$ corresponds to the artificial noise used to exhaust the decoding capabilities of the eavesdropper. Once the decoder successfully decodes the channel and source codewords using its side information $b^{n}$, it can obtain the other part of the key ($k_{1}$) from another bin index of the source codeword, i.e., $v^{n}\left(s_{1},s_{2}\right)\in {\bar{B}}_{2}\left(s_{1},r_{2},k_{1}\right)$. We note that the achievable secret key rate in Equation (5) is a combination of the secret bits transmitted through the noisy channel in the manner of the wiretap channel and the secret bits obtained by the reconstruction of the source at Bob.

The inner bound in [30] is obtained using the *weak* secrecy and uniformity conditions, i.e., $L(c_{n})\leq n\epsilon$ and $U(c_{n})\leq n\epsilon$. However, an improved proof of the inner bound is found in [31], which shows that the *strong* secrecy and uniformity conditions in Equation (4) also hold true. We refer the interested reader to [30,31] for a detailed proof of the inner bound. □

**Remark** **1.** *By setting U=∅, the region in Theorem 1 recovers the results in ([13], Theorems 1 and 4), when the eavesdropper has access to a correlated source, and ([14], Theorem 2), when there is no secret message to be transmitted. The advantage of having two layers of description is that Theorem 1 can potentially achieve higher secret key rates (see Section 4) and it recovers the result of Csiszár and Narayan [8] (see Remark 6).*

**Remark** **2.** *The inner bound in Theorem 1 is a special case of the inner bound recently proposed in [27] for a more general system model.*

**Remark** **3.** *The region in Theorem 1 also recovers the result in ([32], Theorem 1) which was published after the original submission of Bassi et al. [30]. In that work, Alice and Bob communicate over a public noiseless channel of rate $R_{1}$ and a secure noiseless channel of rate $R_{2}$. The proposed achievable scheme in [32] sends the codeword Q through the public channel, i.e., $I\left(Q;Y\right)=R_{1}$, and the codeword T through the secure channel, i.e., $I\left(T;Y|Q\right)=R_{2}$ and I(T;Z|Q)=0. The reader may verify that, by using the aforementioned quantities and η=1, both regions are equal.*

## 3. Main Results

In this section, we first introduce an outer bound for the secret key region (Theorem 2). We then study some special cases where the inner bound of Theorem 1 turns out to achieve the (optimal) secret key region (Propositions 1–3).

### 3.1. Outer Bound

The following theorem gives an outer bound on $R^{⋆}$, i.e., it defines the region $R_{\mathrm{out}}⊇R^{⋆}$.

**Theorem** **2.** *An* outer bound *on the secret key region for this channel model is given by*

(7) $$R_{k}\leq \max_{p\in P}\left\{\eta \left[I(T;Y)-I(T;Z)\right]+I\left(V;B|U\right)-I\left(V;E|U\right)\right\}$$

*subject to*

(8) I(V;A|B)≤ηI(X;Y),

*where P is the set of all input probability distributions given by*

(9) $$P=\left\{\;p(txyzuvabe)=p(tx)p(yz|x)p(abe)p(v|a)p(u|v)\;\right\}$$

*with |T|≤|X|, |U|≤|A|+1, and $\left|V\right|\leq {\left(|A|+1\right)}^{2}$.*

**Proof.**  Refer to Appendix A for details. □

Theorem 2 shows that the secret key generated between Alice and Bob has two components. The first two terms on the r.h.s. of Equation (7) represent the part of the key that is securely transmitted through the noisy channel (given by the random variable *T*) as in the wiretap channel. On the other hand, the last two terms on the r.h.s. of Equation (7) characterize the part of the key that is securely extracted from the correlated sources (given by the random variables *U* and *V*). Since the source and channel variables are independent in the model, it should not be surprising that the variable *T* is independent of (U,V). However, given that the users need to agree on common extracted bits from the source, the noisy channel imposes the restriction in Equation (8) on the amount of information exchanged during that agreement.

**Remark** **4.** *The regions $R_{\mathrm{out}}$ and $R_{\mathrm{in}}$ do not coincide in general. This is due to the presence of the condition in Equation *(6a)* in the inner bound, and the looser condition in Equation *(8)* in the outer bound with respect to Equation *(6b)*. We present in Section 3.2 a few special cases where these differences disappear and both regions coincide.*

**Remark** **5.** *We note that, although the model defines source and channel sequences of potentially different lengths, the final bounds in Equations *(7)* and *(8)* are* single-letter *and they are calculated using the single-letter probability distribution in Equation *(9)*. The difference in sequence length is captured by the coefficient η defined in Equation (4).*

### 3.2. Optimal Characterization of the Secret Key Rate

The inner bound $R_{\mathrm{in}}$ is optimal under certain less-noisy conditions on the channel and/or source components. These special cases are summarized in Table 1 and explained in the sequel.

#### 3.2.1. Eve Has a Less Noisy Channel

If Eve has a less noisy channel than Bob, i.e., $Z{⪰}_{X}\;Y$, the information transmitted over the channel is compromised. Therefore, the amount of secret key that can be generated only depends on the statistical differences between the sources.

**Proposition** **1.** *If $Z{⪰}_{X}\;Y$, a tuple $\left(\eta ,R_{k}\right)\in R_{+}^{2}$ is achievable if and only if there exist random variables U, V, and X on finite sets U, V, and X, respectively, with joint distribution p(uvabexyz)=p(u|v)p(v|a)p(abe)p(x)p(yz|x), which verify*

(10a) $$\begin{array}{c} R_{k}\leq I\left(V;B|U\right)-I\left(V;E|U\right) \end{array}$$

(10b) $$\begin{array}{c} \mathrm{subject}\;\mathrm{to}\;I(V;A|B)\leq \eta \;I(X;Y)\;. \end{array}$$

**Proof.**  Given the less-noisy condition on Eve’s channel, i.e., I(T;Y)≤I(T;Z) for any RV *T* such that T —⚬ X —⚬ (YZ), the bound in Equation (7) is maximized with T=∅. On the other hand, the region in Equation (10) is achievable by setting auxiliary RVs Q=T=X in $R_{\mathrm{in}}$. □

**Remark** **6.** *The secret key capacity of the wiretap channel with a public noiseless channel of rate R ([8], Theorem 2.6) turns out to be a special case of Proposition 1, where X=Y=Z and defining ηH(X)=ηlog|X|≡R.*

#### 3.2.2. Eve Has a Less Noisy Source

If Eve has a less noisy source than Bob, i.e., $E{⪰}_{A}\;B$, the amount of secret key that can be generated depends on the amount of secure information transmitted through the wiretap channel.

**Proposition** **2.** *If $E{⪰}_{A}\;B$, a tuple $\left(\eta ,R_{k}\right)\in R_{+}^{2}$ is achievable if and only if there exist random variables T and X on finite sets T and X, respectively, with joint distribution p(txyz)=p(tx)p(yz|x), which verify*

(11) $$R_{k}\leq \eta \left[I(T;Y)-I(T;Z)\right]\;.$$

**Proof.**  Given the less-noisy condition on Eve’s source, i.e., I(V;B)≤I(V;E) for any RV *V* such that V —⚬ A —⚬ (BE), the bound in Equation (7) is maximized with U=V and independent of the sources. The region in Equation (11) is achievable by using the same auxiliary RVs in the inner bound as in the outer bound. □

**Remark** **7.** *The bound in Equation (11) is equal to the secrecy capacity of the wiretap channel.*

**Remark** **8.** *Even though the bound in Equation (11) becomes independent of the sources sequences $\left(A^{n},B^{n},E^{n}\right)$, we assume n≠0, and thus the rate η is finite.*

#### 3.2.3. Bob Has a Less Noisy Channel and Source

If Bob has a less noisy channel and source than Eve, i.e., $Y{⪰}_{X}\;Z$ and $B{⪰}_{A}\;E$, the lower layers of the channel and source codewords are no longer needed.

**Proposition** **3.** *If $Y{⪰}_{X}\;Z$ and $B{⪰}_{A}\;E$, a tuple $\left(\eta ,R_{k}\right)\in R_{+}^{2}$ is achievable if and only if there exist random variables V and X on finite sets V and X, respectively, with joint distribution p(vabexyz)=p(v|a)p(abe)p(x)p(yz|x), which verify*

(12a) $$\begin{array}{c} R_{k}\leq \eta \left[I(X;Y)-I(X;Z)\right]+I\left(V;B\right)-I\left(V;E\right) \end{array}$$

(12b) $$\begin{array}{c} \text{subject to}\;I(V;A|B)\leq \eta \;I(X;Y)\;. \end{array}$$

**Proof.**  Given the less-noisy conditions on Bob’s channel and source, the bound in Equation (7) is maximized with U=∅ and T=X. The region in Equation (12) is achievable by also setting auxiliary RVs U=Q=∅ and T=X in the inner bound. □

**Remark** **9.** *Proposition 3 extends the results from ([13], Theorem 4) and ([14], Theorem 3) which assume the more stringent conditions of degradedness: A —⚬ B —⚬ E and X —⚬ Y —⚬ Z.*

## 4. Secret Key Agreement over a Wiretap Channel with BEC/BSC Sources

As mentioned in Remark 1, the inner bound introduced in Section 2.2 employs two layers of description, and thus it is an improvement over previously reported results. In this section, we compare the performance of this achievable scheme with the scheme in [13] for a specific binary source and channel model.

### 4.1. System Model

**Figure 2:**
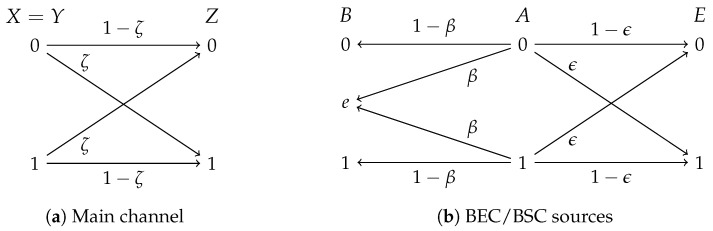
System model for the wiretap channel with BEC/BSC sources.

Consider the communication system depicted in Figure 2. The main channel consists of a noiseless link from Alice to Bob and a binary symmetric channel (BSC) with crossover probability $\zeta \in \left[0,\frac{1}{2}\right]$ from Alice to Eve (see Figure 2a). Additionally, the three nodes have access to correlated sources; in particular, Alice observes a binary uniformly distributed source, i.e., $A∼B\;\left(\frac{1}{2}\right)$, which is the input of two parallel channels, as shown in Figure 2b. Bob observes the output of a binary erasure channel (BEC) with erasure probability β∈[0,1], and Eve, a BSC with crossover probability $\epsilon \in \left[0,\frac{1}{2}\right]$. For simplicity, we assume η=1 in the sequel.

**Remark** **10.** *The sources (A,B,E) satisfy different properties according to the values of the parameters (β,ϵ) [33], specifically:*

- *If 0≤β<2ϵ, E is a* degraded *version of B, i.e., A —⚬ B —⚬ E.*
- *If 2ϵ≤β<4ϵ(1−ϵ), B is* less noisy *than E, i.e., $B{⪰}_{A}\;E$.*
- *If $4\epsilon \left(1-\epsilon \right)\leq \beta <h_{2}\left(\epsilon \right)$, B is* more capable *than E.*

### 4.2. Performance of the Coding Scheme

The following proposition provides a simple expression of the inner bound from Theorem 1. The expression is obtained by simplifying the maximization process of the input distribution, and thus it might not be optimal. However, this suffices to show the higher rates achieved by this scheme as we see later.

**Proposition** **4.** *The tuple $\left(\eta =1,R_{k}\right)\in R_{\mathrm{in}}$ if there exist $u,v,q\in \left[0,\frac{1}{2}\right]$ such that:*

(13a) $$\begin{array}{c} R_{k}\leq \left(1-\beta \right)\left[h_{2}\left(v*u\right)-h_{2}\left(v\right)\right]+h_{2}\left(v*\epsilon \right)-h_{2}\left(v*u*\epsilon \right)+h_{2}\left(\zeta \right)+h_{2}\left(q\right)-h_{2}\left(\zeta *q\right)\;, \end{array}$$

(13b) $$\begin{array}{c} \text{subject to}\;\beta \left[1-h_{2}\left(v*u\right)\right]\leq 1-h_{2}\left(q\right)\;. \end{array}$$

**Proof.**  The bound in Equation (13) is directly calculated from Equations (5) and (6a) with the following choice of input random variables: T=X, $Q=X⊕Q^{\prime }$, $V=A⊕V^{\prime }$, and $U=V⊕U^{\prime }$. Here, $X∼B\;\left(\frac{1}{2}\right)$, $Q^{\prime }∼B\left(q\right)$, $V^{\prime }∼B\left(v\right)$, and $U^{\prime }∼B\left(u\right)$, and each random variable is independent of each other and (A,B,E). The condition in Equation (6b) in the inner bound becomes redundant with the aforementioned choice of input distribution. □

As previously mentioned, we provide next the inner bound presented in ([13], Theorem 4) as a means of comparison. This inner bound is similar to Theorem 1 but with only one layer of description for the source *A*; thus, its achievable region is denoted $R_{\mathrm{in}-1L}$. We note that Theorem 4 from [13] is actually a capacity result assuming that A —⚬ B —⚬ E and X —⚬ Y —⚬ Z. In our present example, only the second Markov chain holds independently of the value of the parameters β and ϵ, but this does not invalidate the use of the inner bound.

**Proposition** **5** ([13], Theorem 4)**.** *The tuple $\left(\eta =1,R_{k}\right)\in R_{\mathrm{in}-1L}$ if and only if*

(14) $$R_{k}\leq {\left[h_{2}\left(\epsilon \right)-\beta \right]}^{+}+h_{2}\left(\zeta \right)\;.$$

**Proof.**  See Appendix B. □

**Remark** **11.** *Proposition 5 is a special case of Proposition 4 with $u=q=\frac{1}{2}$, and v=0 or $v=\frac{1}{2}$. As mentioned in Remark 1, the inner bound ([13], Theorem 4) is a special case of Theorem 1 with U=∅ (thus $u=\frac{1}{2}$). Moreover, given that in this model the Markov chain X —⚬ Y —⚬ Z holds, the channel codebook of Proposition 5 only has one layer (thus $q=\frac{1}{2}$). On the other hand, there are two layers of description in Proposition 4, and whenever U≠∅ (i.e., $u<\frac{1}{2}$), we have that Q≠∅ (i.e., $q<\frac{1}{2}$). This relationship is determined by Equation *(13b)*.*

**Figure 3:**
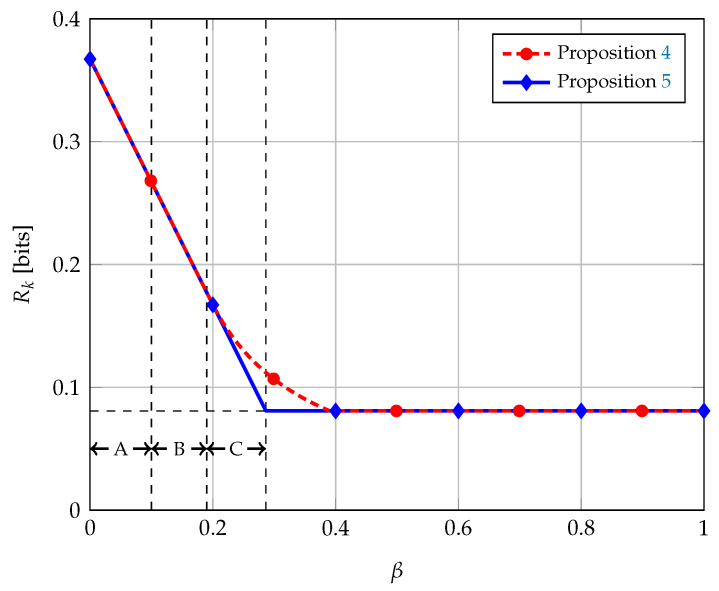
Achievable secret key rates for the wiretap channel with BEC/BSC sources, with ζ=0.01 and ϵ=0.05. In Region A, A —⚬ B —⚬ E; in Region B, $B{⪰}_{A}\;E$; and, in Region C, *B* is more capable than *E*. The horizontal dotted line corresponds to the secrecy capacity of the main channel, i.e., $h_{2}\left(\zeta \right)$.

We performed numerical optimization of the bound in Equation (13) for different values of β while fixing ζ=0.01 and ϵ=0.05; the results are shown in Figure 3 along with the bound in Equation (14). We see in the figure the advantage of having two layers of description for the source *A*. The proposed scheme in Proposition 4 attains higher secret key rates than the scheme with only one layer of description (Proposition 5) for intermediate values of β. It is in this regime, when the source *B* is no longer less noisy than *E*, that two layers of description are needed.

## 5. Summary and Concluding Remarks

In this work, we investigated the problem of secret key generation over a noisy channel in presence of correlated sources (independent of the main channel) at all terminals. We introduced a novel outer bound for this channel model, which allowed us to show that a particular achievable scheme is optimal for all classes of less-noisy sources and channels (Propositions 1–3). In Section 4, we further compared the performance of the aforementioned achievable scheme with a previously reported result for a simple binary model. Numerical computation of the corresponding bounds provided interesting insights on the regimes where the achievable scheme outperforms the previous one.

This work, however, does not address the scenario where the sources and the noisy channel are correlated. The extension of the previously mentioned result of Prabhakaran et al. [14] by using two description layers is a natural consequence. Indeed, this extension—posterior to the short version of the present work in [30]—has been recently addressed in [27]. By using two description layers, the proposed achievable scheme recovers the present inner bound for η=1 provided that the sources are independent of the channel.

## Figures and Tables

**Table 1:** Regimes where Theorem 1 is optimal. No secret key is achievable if $Z{⪰}_{X}\;Y$ and $E{⪰}_{A}\;B$.

$Z{⪰}_{X}\;Y$			$Y{⪰}_{X}\;Z$	
$E{⪰}_{A}\;B$	$B{⪰}_{A}\;E$		$E{⪰}_{A}\;B$	$B{⪰}_{A}\;E$
$R_{k}=0$	Proposition 1		Proposition 2	Proposition 3

## Abbreviations

The following abbreviations are used in this manuscript:

i.i.d.	independent and identically distributed
r.h.s.	right-hand side
w.r.t.	with respect to

## Appendix A. Proof of Theorem 2

The outer bound is derived by following similar steps to those in ([28], Theorem 4) which assumes η=1. It is reproduced here for completeness.

Let $\left(\eta ,R_{k}\right)$ be an achievable tuple according to Definition 2, and ϵ>0. Then, there exists a $\left(2^{nR_{k}},n,m\right)$ secret key code $c_{n}$ with functions φ(·), $\psi_{a}\left(\cdot \right)$, and $\psi_{b}\left(\cdot \right)$ such that

(A1a) $$\begin{array}{cc} X^{m} & =\varphi (A^{n},R_{r})\;, \end{array}$$

(A1b) $$\begin{array}{cc} K\; & =\psi_{a}\left(A^{n},R_{r}\right)\;, \end{array}$$

(A1c) $$\begin{array}{cc} \hat{K}\; & =\psi_{b}\left(B^{n},Y^{m}\right)\;, \end{array}$$

that verify

(A2a) $$\begin{array}{cc} \frac{m}{n} & \leq \eta +\epsilon \;, \end{array}$$

(A2b) $$\begin{array}{cc} \mathrm{Pr}\;\left\{K\neq \hat{K}\right\} & \leq \epsilon \;, \end{array}$$

(A2c) $$\begin{array}{cc} I(K;E^{n}Z^{m}) & \leq \epsilon \;, \end{array}$$

(A2d) $$\begin{array}{cc} nR_{k}-H(K) & \leq \epsilon \;, \end{array}$$

where we have dropped the conditioning on the codebook $c_{n}$ from Equations (A2b)–(A2d) and all subsequent calculations for clarity. Before continuing, we present the following remark that is useful to establish Markov chains between the random variables.

**Remark** **A1.**
*From the fact that random variables $A_{i}$, $B_{i}$, $E_{i}$ are independent across time and the channel X↦(Y,Z) is memoryless and without feedback, the joint distribution of $\left(K,A^{n},B^{n},E^{n},X^{m},Y^{m},Z^{m}\right)$ can be written as follows. For each i∈[1:n] and each j∈[1:m], we have*

(A3) $$\begin{array}{cc} p\left(k,a^{n},b^{n},e^{n},x^{m},y^{m},z^{m}\right)= & p\left(a^{i-1},b^{i-1},e^{i-1}\right)\;p\left(a_{i},b_{i},e_{i}\right)\;p\left(a_{i+1}^{n},b_{i+1}^{n},e_{i+1}^{n}\right)\\ & \;\times p\left(k,x^{m}|a^{n}\right)\;p\left(y^{j-1},z^{j-1}|x^{j-1}\right)\;p\left(y_{j},z_{j}|x_{j}\right)\;p\left(y_{j+1}^{m},z_{j+1}^{m}|x_{j+1}^{m}\right)\;, \end{array}$$

*where $P_{\varphi }\left(x^{m}|a^{n}\right)=\sum_{\forall \;k}p\left(k,x^{m}|a^{n}\right)$ and $P_{\psi_{a}}\left(k|a^{n}\right)=\sum_{\forall \;x^{m}}p\left(k,x^{m}|a^{n}\right)$ are the distributions of the stochastic functions in Equations *(A1a)* and *(A1b)*, respectively.*

We may now carry on with the derivation of the outer bound. First, consider,

(A4a)nRk≤H(K)+ϵ=H(K|EnYm)+I(K;EnYm)+ϵ(A4b)≤H(K|EnYm)+I(K;EnYm)−I(K;EnZm)+2ϵ=H(K|EnYm)+I(K;Ym|En)−I(K;Zm|En)+2ϵ[2](A4c)≤H(K|EnYm)−H(K|BnYm)+I(K;Ym|En)−I(K;Zm|En)+nϵ′(A4d)=I(K;Bn|Ym)−I(K;En|Ym)︸Rs+I(K;Ym|En)−I(K;Zm|En)︸Rc+nϵ′,

where

- Equation (A4a) stems from the uniformity of the keys in Equation (A2d).
- Equation (A4b) is due to the security condition in Equation (A2c).
- Equation (A4c) follows from Equations (A1) and (A2b), and Fano’s inequality, $H(K|B^{n}Y^{m})\leq n\epsilon$.

We now study separately the “source” term $R_{s}$ and the “channel” term $R_{c}$. Hence,

Rs=∑i=1nI(K;Bi|YmBi−1)−I(K;Ei|YmEi+1n)(A5a)=∑i=1nI(K;Bi|YmBi−1Ei+1n)−I(K;Ei|YmBi−1Ei+1n)(A5b)=∑i=1nI(Vi;Bi|Ui)−I(Vi;Ei|Ui)(A5c)=n[I(VJ;BJ|UJJ)−I(VJ;EJ|UJJ)](A5d)=n[I(V;B|U)−I(V;E|U)],

where

- Equation (A5a) is due to Csiszár sum identity.
- Equation (A5b) follows from the definition of the auxiliary RVs $U_{i}=\left(Y^{m}B^{i-1}E_{i+1}^{n}\right)$ and $V_{i}=\left(KU_{i}\right)$.
- Equation (A5c) introduces the auxiliary RV *J* uniformly distributed over [1:n] and independent of all the other variables.
- Equation (A5d) stems from the definition of random variables $U=(U_{J}J)$, $V=(V_{J}J)$, $B=B_{J}$, and $E=E_{J}$.

This establishes the “source” term in Equation (A4d) with auxiliary RVs (U,V) that satisfy the following Markov chain

(A6) $$U_{i}\;—\;\;\;\;\;\;⚬\;V_{i}\;—\;\;\;\;\;\;⚬\;A_{i}\;—\;\;\;\;\;\;⚬\;\left(B_{i}E_{i}\right)\;.$$

The first part of Equation (A6) is trivial given the definition $V_{i}=\left(KU_{i}\right)$, whereas the second part follows from the i.i.d. nature of the sources and that they are correlated to the main channel only through the encoder’s input in Equation (A1a), see Equation (A3),

(A7) $$\left(KY^{m}B^{i-1}E_{i+1}^{n}\right)\;—\;\;\;\;\;\;⚬\;A_{i}\;—\;\;\;\;\;\;⚬\;\left(B_{i}E_{i}\right)\;.$$

The “channel” term $R_{c}$ can be single-letterized similarly,

(A8) $$R_{c}=m\left[I\left(T;Y|Q\right)-I\left(T;Z|Q\right)\right]\;,$$

where we first define the auxiliary RVs $Q_{i}=\left(E^{n}Y^{i-1}Z_{i+1}^{m}\right)$ and $T_{i}=\left(KQ_{i}\right)$, we then introduce the auxiliary RV *L* uniformly distributed over [1:m], and we finally define $Q=(Q_{L}L)$, $T=(T_{L}L)$, $Y=Y_{L}$, and $Z=Z_{L}$. The auxiliary RVs in this term, i.e., (Q,T), satisfy the following Markov chain

(A9) $$Q_{i}\;—\;\;\;\;\;\;⚬\;T_{i}\;—\;\;\;\;\;\;⚬\;X_{i}\;—\;\;\;\;\;\;⚬\;\left(Y_{i}Z_{i}\right)\;,$$

where the nontrivial part is due to the memoryless property of the channel and (A1b), provided the joint probability distribution satisfies Equation (A3). Since neither *Q* nor *T* appears on other parts of the outer bound, we may expand $R_{c}$ as

(A10) $$\begin{array}{cc} R_{c} & =m\sum_{q\in Q}p_{Q}\left(q\right)\left[I(T;Y|Q=q)-I(T;Z|Q=q)\right]\\ & \leq m\max_{q\in Q}\left[I(T;Y|Q=q)-I(T;Z|Q=q)\right]\\ & =m[I\left(T^{⋆};Y\right)-I\left(T^{⋆};Z\right)]\;, \end{array}$$

where in the last step we choose auxiliary RV $T^{⋆}∼p_{T|Q}\left(\cdot |q\right)$.

Gathering Equations (A4), (A5), (A8), and (A10), the rate of the secret key writes

(A11) $$R_{k}\leq I\left(V;B|U\right)-I\left(V;E|U\right)+\frac{m}{n}\left[I(T;Y)-I(T;Z)\right]+\epsilon^{\prime }.$$

If we let (n,m)→∞ and take arbitrarily small $\epsilon^{\prime }$, we obtain the bound in Equation (7).

To obtain Equation (8), we use the following Markov chain that is a consequence of Equation (A1a), provided the joint probability satisfies Equation (A3):

(A12) $$\left(B^{n}E^{n}\right)\;—\;\;\;\;\;\;⚬\;A^{n}\;—\;\;\;\;\;\;⚬\;X^{m}\;—\;\;\;\;\;\;⚬\;\left(Y^{m}Z^{m}\right)\;.$$

Due to the data processing inequality, we have

(A13) $$I\left(A^{n};Y^{m}\right)\leq I\left(X^{m};Y^{m}\right)\leq m\;I\left(X;Y\right)\;,$$

where in the last inequality we use the memoryless property of the channel. Next, consider

(A14a)I(An;Ym)=I(AnBn;Ym)≥I(An;Ym|Bn)=I(An;KYm|Bn)−I(An;K|BnYm)(A14b)≥I(An;KYm|Bn)−nϵ(A14c)≥n[I(A;V|B)−ϵ],

where

- Equation (A14a) follows from the Markov chain in Equation (A12).
- Equation (A14b) stems from $H(K|B^{n}Y^{m})\leq n\epsilon$ due to Equations (A1) and (A2b), and $H(K|A^{n}B^{n}Y^{m})\geq 0$.

For the last step, i.e., Equation (A14c), consider

(A15a)I(KYm;An|Bn)=I(KYm;AnEn|Bn)=∑i=1nI(KYm;AiEi|BnAi+1nEi+1n)(A15b)≥∑i=1nI(KYmBi−1Ei+1n;AiEi|Bi)(A15c)=∑i=1nI(Vi;AiEi|Bi)≥∑i=1nI(Vi;Ai|Bi)[2](A15d)=nI(VJ;AJ|BJJ)(A15e)=nI(VJJ;AJ|BJ)(A15f)=nI(V;A|B),

where

- Equation (A15a) stems from the Markov chain $\left(B^{n}E^{n}\right)\;—\;\;\;\;\;\;⚬\;A^{n}\;—\;\;\;\;\;\;⚬\;\left(KY^{m}\right)$.
- Equation (A15b) follows from the sources being i.i.d., i.e., $\left(A_{i}E_{i}\right)\;—\;\;\;\;\;\;⚬\;B_{i}\;—\;\;\;\;\;\;⚬\;\left(B^{i-1}B_{i+1}^{n}A_{i+1}^{n}E_{i+1}^{n}\right)$.
- Equation (A15c) is due to the auxiliary RV $V_{i}=\left(KY^{m}B^{i-1}E_{i+1}^{n}\right)$.
- Equation (A15d) introduces the auxiliary RV *J* uniformly distributed over [1:n] and independent of all the other variables.
- Equation (A15e) follows from the independence of *J* and $\left(A_{J}B_{J}\right)$.
- Equation (A15f) stems from the definition of random variables $V=(V_{J}J)$, $B=B_{J}$, and $A=A_{J}$.

Putting Equations (A13) and (A14) together, we obtain:

(A16) $$I\left(V;A|B\right)\leq \frac{m}{n}I\left(X;Y\right)+\epsilon \;,$$

which gives the condition in Equation (8) as we let (n,m)→∞ and take an arbitrarily small ϵ.

Although the definition of the auxiliary RVs (TUV) used in the proof makes them arbitrarily correlated, the bounds in Equations (7) and (8) only depend on the *marginal* PDs p(tx) and p(uv|a). Consequently, we can restrict the set of possible joint PDs to Equation (9), i.e., independent source and channel variables, and still achieve the maximum.

The bounds on the cardinality of the alphabets T, U, and V for the auxiliary RVs follow from Fenchel–Eggleston–Carathéodory’s theorem and the standard cardinality bounding technique ([29], Appendix C); therefore, their proof is omitted. This concludes the proof of Theorem 2. □

## Appendix B. Proof of Proposition 5

For completeness, we first present the inner bound from ([13], Theorem 4) but rewritten using the notation of the present work:

(A17a) $$\begin{array}{c} R_{k}\leq \max_{p(x)p(v|a)}\left\{I(V;B)-I(V;E)+\eta \;I(X;Y|Z)\right\} \end{array}$$

(A16b) $$\begin{array}{c} \text{subject to}\;I(V;A|B)\leq \eta \;I(X;Y)\;. \end{array}$$

In the sequel, we assume η=1.

The main channel in the system model depicted in Figure 2a is not only degraded but also *Y* equals *X*; thus, the last term on the r.h.s. of Equation (A17a) may be expanded as follows

(A18) I(X;Y|Z)=H(X|Z)=H(X)+H(Z|X)−H(Z).

Since *X* is the input of a BSC of parameter ζ and output *Z*, it is clear that

(A19) $$I\left(X;Y|Z\right)\leq H\left(Z|X\right)=h_{2}\left(\zeta \right)\;,$$

with equality if and only if $X∼B\left(\frac{1}{2}\right)$. Furthermore, this choice of *X* maximizes the r.h.s. of Equation (A17b) and makes the condition redundant:

(A20) I(V;A|B)≤H(A|B)=βH(A)=β≤1=H(X),

given that $A∼B\;\left(\frac{1}{2}\right)$ and 0≤β≤1.

It remains to be determined what the maximizing value of the first two terms on the r.h.s. of Equation (A17a) is. Let us first assume that *B* is *more capable* than *E*, i.e., $0\leq \beta <h_{2}\left(\epsilon \right)$ according to Remark 10. Then, we may write

I(V;B)−I(V;E) =I(A;B)−I(A;E)−I(A;B|V)−I(A;E|V)(A21a) ≤I(A;B)−I(A;E)(A21b) =H(A|E)−H(A|B)(A21c) =h2(ϵ)−β,

where the inequality is due to I(A;B|V)≥I(A;E|V) for all p(v,a) given the more capable assumption. The bound in Equation (A21) holds with equality if and only if V=A. We also note that Equation (A21) is a monotonically decreasing function of β and it is zero when $\beta =h_{2}\left(\epsilon \right)$. For $\beta >h_{2}\left(\epsilon \right)$, the bound in Equation (A21) is no longer valid; however, we can rightfully argue that, as Bob’s source degrades while Eve’s remains the same, it is not possible to obtain more secret bits from the sources than for $\beta =h_{2}\left(\epsilon \right)$. Therefore, for $\beta >h_{2}\left(\epsilon \right)$,

(A22) I(V;B)−I(V;E)≤0,

which holds with equality if and only if V=∅.

Combining Equations (A17), (A19), (A21), and (A22), we obtain the bound in Equation (14). This concludes the proof of Proposition 5. □

## References

[1] Wyner A.D. (1975). The Wire-Tap Channel. Bell Syst. Tech. J..

[2] Csiszár I., Körner J. (1978). Broadcast Channels with Confidential Messages. IEEE Trans. Inf. Theory.

[3] Shannon C.E. (1949). Communication Theory of Secrecy Systems. Bell Syst. Tech. J..

[4] Chorti A., Hollanti C., Belfiore J.C., Poor H.V., Baldi M., Tomasin S. (2016). Physical Layer Security: A Paradigm Shift in Data Confidentiality. Physical and Data-Link Security Techniques for Future Communication Systems.

[5] Narayan P., Tyagi H. (2016). Multiterminal Secrecy by Public Discussion. Foundations and Trends® in Communications and Information Theory.

[6] Maurer U.M. (1993). Secret Key Agreement by Public Discussion from Common Information. IEEE Trans. Inf. Theory.

[7] Ahlswede R., Csiszár I. (1993). Common Randomness in Information Theory and Cryptography—Part I: Secret Sharing. IEEE Trans. Inf. Theory.

[8] Csiszár I., Narayan P. (2000). Common Randomness and Secret Key Generation with a Helper. IEEE Trans. Inf. Theory.

[9] Csiszár I., Narayan P. (2008). Secrecy Capacities for Multiterminal Channel Models. IEEE Trans. Inf. Theory.

[10] Csiszár I., Narayan P. (2013). Secrecy Generation for Multiaccess Channel Models. IEEE Trans. Inf. Theory.

[11] Gohari A.A., Anantharam V. (2010). Information-Theoretic Key Agreement of Multiple Terminals—Part I. IEEE Trans. Inf. Theory.

[12] Gohari A.A., Anantharam V. (2010). Information-Theoretic Key Agreement of Multiple Terminals—Part II: Channel Model. IEEE Trans. Inf. Theory.

[13] Khisti A., Diggavi S.N., Wornell G.W. (2012). Secret-Key Generation Using Correlated Sources and Channels. IEEE Trans. Inf. Theory.

[14] Prabhakaran V.M., Eswaran K., Ramchandran K. (2012). Secrecy via Sources and Channels. IEEE Trans. Inf. Theory.

[15] Bunin A., Piantanida P., Shamai S. The Gaussian Wiretap Channel with Correlated Sources at the Terminals: Secret Communication and Key Generation. Proceedings of the 2016 ICSEE International Conference on the Science of Electrical Engineering.

[16] Salimi S., Skoglund M., Golic J.D., Salmasizadeh M., Aref M.R. (2013). Key Agreement over a Generalized Multiple Access Channel Using Noiseless and Noisy Feedback. IEEE J. Sel. Areas Commun..

[17] Tyagi H. (2013). Common Information and Secret Key Capacity. IEEE Trans. Inf. Theory.

[18] Hayashi M., Tyagi H., Watanabe S. (2016). Secret Key Agreement: General Capacity and Second-Order Asymptotics. IEEE Trans. Inf. Theory.

[19] Courtade T.A., Halford T.R. (2016). Coded Cooperative Data Exchange for a Secret Key. IEEE Trans. Inf. Theory.

[20] Boche H., Schaefer R.F., Poor H.V. On the Computability of the Secret Key Capacity under Rate Constraints. Proceedings of the 2019 IEEE International Conference on Acoustics, Speech and Signal Processing (ICASSP).

[21] Chen Y., Vinck A.J.H. (2008). Wiretap Channel with Side Information. IEEE Trans. Inf. Theory.

[22] Liu W., Chen B. Wiretap Channel with Two-Sided Channel State Information. Proceedings of the 2007 41st Asilomar Conference on Signals, Systems and Computers (ACSSC).

[23] Gelfand S.I., Pinsker M.S. (1980). Coding for Channel with Random Parameters. Probl. Control Inf. Theory.

[24] Muramatsu J. General Formula for Secrecy Capacity of Wiretap Channel with Noncausal State. Proceedings of the 2014 IEEE International Symposium on Information Theory (ISIT).

[25] Goldfeld Z., Cuff P., Permuter H.H. (2016). Wiretap Channels with Random States Non-Causally Available at the Encoder. arXiv.

[26] Zibaeenejad A. (2015). Key Generation over Wiretap Models with Non-Causal Side Information. IEEE Trans. Inf. Forensics Secur..

[27] Bunin A., Goldfeld Z., Permuter H.H., Shamai S., Cuff P., Piantanida P. (2018). Key and Message Semantic-Security over State-Dependent Channels. IEEE Trans. Inf. Forensics Secur..

[28] Bassi G., Piantanida P., Shamai S. (2019). The Wiretap Channel with Generalized Feedback: Secure Communication and Key Generation. IEEE Trans. Inf. Theory.

[29] El Gamal A., Kim Y.H. (2011). Network Information Theory.

[30] Bassi G., Piantanida P., Shamai S. Secret Key Generation over Noisy Channels with Common Randomness. Proceedings of the 2016 IEEE International Symposium on Information Theory (ISIT).

[31] Bassi G., Piantanida P., Shamai S. (2016). Secret Key Generation over Noisy Channels with Correlated Sources. arXiv.

[32] Cao D., Kang W. Secret key generation from correlated sources and secure link. Proceedings of the 2017 9th International Conference on Wireless Communications and Signal Processing (WCSP).

[33] Nair C. Capacity Regions of Two New Classes of 2-Receiver Broadcast Channels. Proceedings of the 2009 IEEE International Symposium on Information Theory (ISIT).

